# Local Local Reasoning: A BI-Hyperdoctrine for Full Ground Store

**DOI:** 10.1007/978-3-030-45231-5_28

**Published:** 2020-04-17

**Authors:** Miriam Polzer, Sergey Goncharov

**Affiliations:** 8 ENS/CNRS, Université Paris-Saclay, Cachan, France; 9 University of Duisburg-Essen, Duisburg, Germany; FAU Erlangen-Nürnberg, Erlangen, Germany

## Abstract

Modelling and reasoning about dynamic memory allocation is one of the well-established strands of theoretical computer science, which is particularly well-known as a source of notorious challenges in semantics, reasoning, and proof theory. We capitalize on recent progress on categorical semantics of *full ground store*, in terms of a *full ground store monad*, to build a corresponding semantics of a higher order logic over the corresponding programs. Our main result is a construction of an *(intuitionistic) BI-hyperdoctrine*, which is arguably the semantic core of higher order logic over local store. Although we have made an extensive use of the existing generic tools, certain principled changes had to be made to enable the desired construction: while the original monad works over total heaps (to disable dangling pointers), our version involves partial heaps (*heaplets*) to enable compositional reasoning using separating conjunction. Another remarkable feature of our construction is that, in contrast to the existing generic approaches, our BI-algebra does not directly stem from an internal categorical partial commutative monoid.

## 1 Introduction

Modelling and reasoning about dynamic memory allocation is a sophisticated subject in denotational semantics with a long history (e.g. [19,15,14,16]). Denotational models for dynamic references vary over a large spectrum, and in fact, in two dimensions: depending on the expressivity of the features being modelled (*ground store* – *full ground store* – *higher order store*) and depending on the amount of *intensional* information included in the model (*intensional* – *extensional*), using the terminology of Abramsky [1].

Recently, Kammar et al [9] constructed an extensional monad-based denotational model of the *full ground store*, i.e. permitting not only memory allocation for discrete values, but also storing mutually linked data. The key idea of the latter work is an explicit delineation between the target presheaf category $[\mathbf{W}, \mathbf{Set}]$ on which the full ground store monad acts, and an auxiliary presheaf category $[\mathbf{E}, \mathbf{Set}]$ of *initializations*, naturally hosting a *heap functor* $H$. The latter category also hosts a *hiding monad* $P$, which can be loosely understood as a semantic

* Sergey Goncharov acknowledges support by German Research Foundation (DFG) under project GO 2161/1-2.

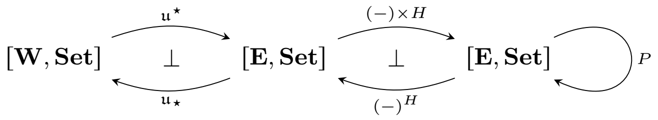

Fig. 1: Construction of the full ground store monad.

mechanism for idealized garbage collection. The full ground store monad is then assembled according to the scheme given in Fig. 1. As a slogan: the *local* store monad is a *global* store monad transform of the hiding monad sandwiched within a geometric morphism.

The fundamental reason, why extensional models of local store involve intricate constructions, such as presheaf categories is that the desirable program equalities include

$$\mathsf{let}\ \ell := \mathsf{new}\, v;\ \ell' := \mathsf{new}\, w\, \mathsf{in}\, p \;=\; \mathsf{let}\ \ell' := \mathsf{new}\, w;\ \ell := \mathsf{new}\, v\, \mathsf{in}\, p \qquad (\ell \not\equiv \ell')$$
$$\mathsf{let}\ \ell := \mathsf{new}\, v\, \mathsf{in}\, \mathsf{ret}\,\star \;=\; \mathsf{ret}\,\star$$
$$\mathsf{let}\ \ell := \mathsf{new}\, v\, \mathsf{in}\ (\mathsf{if}\ \ell = \ell'\ \mathsf{then\ true\ else\ false}) \;=\; \mathsf{false} \qquad (\ell \not\equiv \ell')$$

and these jointly do not have set-based models over countably infinite sets of locations [23, Proposition 6]. The first equation expresses irrelevance of the memory allocation order, the second expresses the fact that an unused cell is always garbage collected and the third guarantees that allocation of a fresh cell does indeed produce a cell different from any other. The aforementioned construction validates these equations and enjoys further pleasant properties, e.g. soundness and adequacy of a higher order language with user defined storable data structures.

The goal of our present work is to complement the semantics of programs over local store with a corresponding principled semantics of *higher order logic*. In order to be able to specify and reason modularly about local store, more specifically, we seek a model of higher order *separation logic* [21]. It has been convincingly argued in previous work on categorical models of separation logic [2,3] that a core abstraction device unifying such models is a notion of *BI-hyperdoctrine*, extending Lawvere's hyperdoctrines [10], which provide a corresponding abstraction for the first order logic. BI-hyperdoctrines are standardly built on *BI-algebras*, which are also standardly constructed from *partial commutative monoids (pcm)*, or more generally from *resource algebras* as in the Iris state of the art advanced framework for higher order separation logic [8]. One subtlety our construction reveals is that it does not seem to be possible to obtain a *BI-algebra* following general recipes from a pcm (or a resource algebra), due to the inherent local nature of the storage model, which does not allow one to canonically map store contents into a global address space. Another subtlety is that the devised logic is necessarily non-classical, which is intuitively explained by the fact that the semantics of programs must be suitably irrelevant to garbage collection, and in our case this follows from entirely formal considerations (Yoneda lemma). It is also worth mentioning that for this reason the logical theory that we obtain is incompatible with the standard (classical or intuitionistic) predicate logic. E.g. the formula $\exists \ell.\, \ell \hookrightarrow 5$ is always valid in our setup, which expresses the fact that a heap *potentially* contains a cell equal to 5 (which need not be reachable) – this is in accord with the second equation above – and correspondingly, the formula $\forall \ell.\, \neg(\ell \hookrightarrow 5)$ is unsatisfiable. This and other similar phenomena are explained by the fact that our semantics essentially behaves as a Kripke semantics along two orthogonal axes: (proof relevant) *cell allocation* and (proof irrelevant) *cell accessibility*. While the latter captures a *programming* view of locality, the latter captures a *reasoning* view of locality, and as we argue (e.g. Example 26), they are generally mutually irreducible.

**Related previous work** As we already pointed out, we take inspiration from the recent categorical approaches to modelling program semantics for dynamic references [9], as well as from higher order separation logic semantic frameworks [2]. Conceptually, the problem of combining separation logic with garbage collection mechanisms goes back to Reynolds [20], who indicated that standard semantics of separation logic in not compatible with garbage collection, which we also reinforce with our construction. Calcagno et al [4] addressed this issue by providing two models. The first model is based on total heaps, featuring the aforementioned effect of "potential" allocations. To cope with heap separation the authors introduced another model based on partial heaps, in which this effect again disappears, and has to be compensated by syntactic restrictions on the assertion language.

**Plan of the paper** After preliminaries (Section 2), we give a modified presentation of a call-by-value language with full ground references and the full ground store monad (Sections 3 and 4) following the lines of [9]. In Section 5 we provide some general results for constructing semantics of higher order separation logics. The main development starts in Section 6 where we provide a construction of a BI-hyperdoctrine. We show some example illustrating our semantics in Section 7 and draw conclusions in Section 8.

## 2 Preliminaries

We assume basic familiarity with the elementary concepts of category theory [12,6], all the way up to monads, toposes, (co)ends and Kan extensions. We denote by $|\mathbf{C}|$ the class of objects of a category $\mathbf{C}$; we often suppress subscripts of natural transformation components if no confusion arises.

In this paper, we work with special kinds of *covariant presheaf toposes*, i.e. functor categories of the form $[\mathbf{C}, \mathbf{Set}]$, where $\mathbf{C}$ is small and satisfies the following *amalgamation condition*: for any $f\colon a \to b$ and $g\colon a \to c$ there exist $g'\colon b \to d$ and $f'\colon c \to d$ such that $f' \circ g = g' \circ f$. Such toposes are particularly well-behaved, and fall into the more general class of *De Morgan* toposes [7]. As presheaf toposes, De Morgan toposes are precisely characterized by the condition

$$\textbf{(put)}\ \frac{\Gamma \vdash_{\mathsf{v}} \ell\colon \mathsf{Ref}_S \qquad \Gamma \vdash_{\mathsf{v}} v\colon \mathsf{CType}(S)}{\Gamma \vdash_{\mathsf{c}} \ell := v\colon 1} \qquad \textbf{(get)}\ \frac{\Gamma \vdash_{\mathsf{v}} \ell\colon \mathsf{Ref}_S}{\Gamma \vdash_{\mathsf{c}} !\ell\colon \mathsf{CType}(S)}$$

$$\textbf{(new)}\ \frac{\begin{array}{c}\Gamma, \ell_1\colon \mathsf{Ref}_{S_1}, \dots, \ell_n\colon \mathsf{Ref}_{S_n} \vdash_{\mathsf{v}} v_1\colon \mathsf{CType}(S_1)\\ \vdots \\ \Gamma, \ell_1\colon \mathsf{Ref}_{S_1}, \dots, \ell_n\colon \mathsf{Ref}_{S_n} \vdash_{\mathsf{v}} v_n\colon \mathsf{CType}(S_n)\\ \Gamma, \ell_1\colon \mathsf{Ref}_{S_1}, \dots, \ell_n\colon \mathsf{Ref}_{S_n} \vdash_{\mathsf{c}} p\colon A\end{array}}{\Gamma \vdash_{\mathsf{c}} \mathsf{letref}\, \ell_1 := v_1, \dots, \ell_n := v_n \,\mathsf{in}\, p\colon A}$$

Fig. 2: Term formation rules for memory management constructs.

that $2 = 1 + 1$ is a retract of the subobject classifier $\Omega$. More specifically, our $\mathbf{C}$ support further useful structure, in particular, a strict monoidal tensor $\oplus$ with jointly epic injections $\mathsf{in}_1$, $\mathsf{in}_2$, forming an *independent coproduct* structure, as recently identified by Simpson [22]. Moreover, if the coslices $c \downarrow \mathbf{C}$ support independent products, we obtain *local independent coproducts* in $\mathbf{C}$, which are essentially cospans $c_1 \to c_1 \oplus_c c_2 \leftarrow c_2$ in $c \downarrow \mathbf{C}$. Given $\rho_1\colon c \to c_1$ and $\rho_2\colon c \to c_2$, we thus always have $\rho_1 \bullet \rho_2\colon c_1 \to c_1 \oplus_c c_2$ and $\rho_2 \bullet \rho_1\colon S_2 \to c_1 \oplus_c c_2$, such that $(\rho_1 \bullet \rho_2) \circ \rho_1 = (\rho_2 \bullet \rho_1) \circ \rho_2$, and as a consequence, $[\mathbf{C}, \mathbf{Set}]$ is a De Morgan topos. Intuitively, the category $\mathbf{C}$ represents worlds in the sense of *possible world semantics* [15,19]. A morphism $\rho\colon a \to b$ witnesses the fact that $b$ is a *future* world w.r.t. $a$. Existence of local independent products intuitively ensures that diverse futures of a given world can eventually be unified in a canonical way.

Every functor $\mathfrak{f}\colon \mathbf{C} \to \mathbf{D}$ induces a functor $\mathfrak{f}^\star\colon [\mathbf{D}, \mathbf{Set}] \to [\mathbf{C}, \mathbf{Set}]$ by precomposition with $\mathfrak{f}$. By general considerations, there is a right adjoint $\mathfrak{f}_\star\colon [\mathbf{C}, \mathbf{Set}] \to [\mathbf{D}, \mathbf{Set}]$, computed as $\mathsf{Ran}_{\mathfrak{f}}$, the right Kan extension along $\mathfrak{f}$. This renders the adjunction $\mathfrak{f}^\star \dashv \mathfrak{f}_\star$, as a *geometric morphism*, in particular, $\mathfrak{f}^\star$ preserves all finite limits.

## 3 A Call-by-Value Language with Local References

To set the context, we consider the following higher order language of programs with local references by slightly adapting the language of Kammar et al [9] to match with the *fine-grain call-by-value* perspective [11]. This allows us to formally distinguish *pure* and *effectful* judgements. First, we postulate a collection of *cell sorts* $\mathcal{S}$ and then introduce further types with the grammar:

$$A, B \ldots ::= 0 \mid 1 \mid A \times B \mid A + B \mid A \to B \mid \mathsf{Ref}_S \qquad (S \in \mathcal{S}) \tag{1}$$

A type is *first order* if it does not involve the function type constructors $A \to B$. We then fix a map $\mathsf{CType}$, assigning a first order type to every given sort from $\mathcal{S}$. We show three term formation rules over these data in Fig. 2 specific to local store.

Here the $\mathsf{v}$-indices at the turnstiles indicate *values* and the $\mathsf{c}$-indices indicate *computations*. In **(put)** the cell referenced by $\ell$ is updated with a value $v$, **(get)** returns a value under the reference $\ell$ and **(new)** simultaneously allocates new cells filled with the values $v_1, \ldots, v_n$ and makes them accessible in $p$ under the corresponding references $\ell_1, \ldots, \ell_n$. A fine-grain call-by-value language is interpreted standardly in a category with a monad, which in our case must additionally provide a semantics to the rules **(put)**, **(get)** and **(new)**. We present this monad in detail in the next section.

**Example 1 (Doubly Linked Lists).** Let $\mathcal{S} = \{\mathit{DLList}\}$ and let $\mathsf{CType}(\mathit{DLList}) = 2 \times (\mathsf{Ref}_{\mathit{DLList}} + 1) \times (\mathsf{Ref}_{\mathit{DLList}} + 1)$, which indicates that a list element is a Boolean (i.e. an element of $2 = 1 + 1$) and two pointers (forwards and backwards) to list elements, each of which may be missing. Note that we thus avoid empty lists and null-pointers: every list contains at least one element, and the elements added by $+1$ cannot be dereferenced. This example provides a suitable illustration for the $\mathsf{letref}$ construct. E.g. the program

$$\mathsf{letref}\,\ell_1 \mathrel{:=} (0, \mathsf{inr}\,\star, \mathsf{inl}\,\ell_2); \ell_2 \mathrel{:=} (1, \mathsf{inl}\,\ell_1, \mathsf{inr}\,\star)\;\mathsf{in}\;\mathsf{ret}\,\ell_1$$

simultaneously creates two list elements pointing to each other and returns a reference to the first one.

## 4 Full Ground Store in the Abstract

We proceed to present the full ground store monad by slightly tweaking the original construction [9] towards higher generality. The main distinction is that we do not recur to any specific program syntax and proceed in a completely axiomatic manner in terms of functors and natural transformations. This mainly serves the purpose of developing our logic in Section 6, which will require a coherent upgrade of the present model. Besides this, in this section we demonstrate flexibility of our formulation by showing that it also instantiates to the model previously developed by Plotkin and Power [16] (Theorem 8).

Our present formalization is parametric in three aspects: the set of *sorts* $\mathcal{S}$, the set of *locations* $\mathcal{L}$ and a map $\mathsf{range}$, introduced below for interpreting $\mathcal{S}$. We assume that $\mathcal{L}$ is canonically isomorphic to the set of natural numbers $\mathbb{N}$ under $\#\colon \mathcal{L} \cong \mathbb{N}$. Using this isomorphism, we commonly use the "shift of $\ell \in \mathcal{L}$ by $n \in \mathbb{N}$", defined as follows: $\ell + n = \#^{-1}(\#\ell + n)$.

**Heap layouts and abstract heap(let)s** Let $\mathbf{W}$ be a category of *(heap) layouts* and injections defined as follows: an object $w \in |\mathbf{W}|$ is a finitely supported partial function $w\colon \mathcal{L} \rightharpoonup_{\mathit{fin}} \mathcal{S}$ and a morphism $\rho\colon w \to w'$ is a type preserving injection $\rho\colon \mathsf{dom}\, w \to \mathsf{dom}\, w'$, i.e. for all $l \in \mathsf{img}\, w$, $w(\ell) = w'(\rho(\ell))$. We will equivalently view $w$ as a left-unique subset of $\mathcal{L} \times \mathcal{S}$ and hence use the notation $(\ell\colon S) \in w$ as an equivalent of $w(\ell) = S$. Injections $\rho\colon w \to w'$ with the property that $w(\ell\colon S) = \ell\colon S$ for all $(\ell\colon S) \in w$ we also call *inclusions* and write $w \subseteq w'$ instead of $\rho\colon w \to w'$, for obviously there is at most one inclusion from $w$ to $w'$. If $w \subseteq w'$

then we call $w$ a *sublayout* of $w'$. We next postulate

$$\mathsf{range}\colon \mathcal{S} \to [\mathbf{W}, \mathbf{Set}].$$

The idea is, given a sort $S \in \mathcal{S}$ and a heap layout $w \in |\mathbf{W}|$, $\mathsf{range}(S)(w)$ yields the set of possible values for cells of type $S$ over $w$.

**Example 2.** Assuming the grammar (1) and a corresponding map $\mathsf{CType}$, a generic type $A$ is interpreted as a presheaf $\underline{A}\colon \mathbf{W} \to \mathbf{Set}$, by obvious structural induction, e.g. $\underline{A \times B} = \underline{A} \times \underline{B}$, except for the clause for $\mathsf{Ref}$, for which $(\underline{\mathsf{Ref}_S})w = w^{-1}(S)$. This yields the following definition for $\mathsf{range}$: $\mathsf{range}(S) = \underline{\mathsf{CType}(S)}$ [9].

**Example 3 (Simple Store).** By taking $\mathcal{S} = \{\star\}$, $\mathcal{L} = \mathbb{N}$ (natural numbers) and $\mathsf{range}(\star)(w) = \mathcal{V}$ where $\mathcal{V}$ is a fixed set of *values*, we essentially obtain the model previously explored by Plotkin and Power [16]. We reserve the term *simple store* for this instance. Simple store is a ground store (since $\mathsf{range}$ is a constant functor), moreover this store is untyped (since $\mathcal{S} = \{\star\}$) and the locations $\mathcal{L}$ are precisely the natural numbers.

A *heap* over a layout $w$ assigns to each $(\ell\colon S) \in w$ an element from $\mathsf{range}(S)(w)$. More generally, a *heaplet* over $w$ assigns an element from $\mathsf{range}(S)(w)$ to *some*, possibly not all, $(\ell\colon S) \in w$. We thus define the following *heaplet bi-functor* $\mathcal{H}\colon \mathbf{W}^{\mathsf{op}} \times \mathbf{W} \to \mathbf{Set}$:

$$\mathcal{H}(w^-, w^+) = \prod\nolimits_{(\ell\colon S) \in w^-} \mathsf{range}(S)(w^+)$$

and identify the elements of $\mathcal{H}(w^-, w^+)$ with heaplets and the elements of $\mathcal{H}(w, w)$ with heaps. Of course, we intend to use $\mathcal{H}(w^-, w^+)$ for such $w^-$ and $w^+$ that the former is a sublayout of the latter. The contravariant action of $H$ is given by projection and the covariant action is induced by functoriality of $\mathsf{range}(S)$.

$$\begin{aligned}
\mathsf{pr}_{(\ell\colon S)}(\mathcal{H}(w^-, \rho_1\colon w_1^+ \to w_2^+)(\eta \in \mathcal{H}(w^-, w_1^+))) &= \mathsf{range}(S)(\rho_1)(\mathsf{pr}_{(\ell\colon S)}\,\eta)\\
\mathsf{pr}_{(\ell\colon S)}(\mathcal{H}(\rho_2\colon w_2^- \to w_1^-, w^+)(\eta \in \mathcal{H}(w_1^-, w^+))) &= \mathsf{pr}_{\rho_2(\ell\colon S)}\,\eta
\end{aligned}$$

The heaplet functor preserves independent coproduct, we overload the $\oplus$ operation with the isomorphism $\oplus\colon \mathcal{H}(w_1, w) \times \mathcal{H}(w_2, w) \cong \mathcal{H}(w_1 \oplus w_2, w)$.

**Example 4.** For illustration, consider the following simplistic example. Let $\mathcal{S} = \{\mathit{Int}, \mathsf{Ref}_{\mathit{Int}}, \mathsf{Ref}_{\mathsf{Ref}_{\mathit{Int}}}, \dots\}$ where $\mathit{Int}$ is meant to capture the ground type of integers and recursively, $\mathsf{Ref}_A$ is the type of pointers to $A$. Then, we put

$$\mathsf{range}(\mathit{Int})(w) = \mathbb{Z}, \qquad \mathsf{range}(\mathsf{Ref}_S)(w) = w^{-1}(S) = \{\ell \in \mathsf{dom}\, w \mid w(\ell) = S\}.$$

For a heaplet example, consider $w^- = \{\ell_1\colon \mathit{Int}, \ell_2\colon \mathsf{Ref}_{\mathit{Int}}\}$ and $w^+ = \{\ell_1\colon \mathit{Int}, \ell_2\colon \mathsf{Ref}_{\mathit{Int}}, \ell_3\colon \mathit{Int}\}$. Hence, $w^-$ is a sublayout of $w^+$. By viewing the elements of $\mathcal{H}(w^-, w^+)$ as lists of assignments on $w^-$, we can define $s_1, s_2 \in \mathcal{H}(w^-, w^+)$ as follows: $s_1 = [\ell_1\colon \mathit{Int} \mapsto 5,\ \ell_2\colon \mathsf{Ref}_{\mathsf{int}} \mapsto \ell_1]$, $s_2 = [\ell_1\colon \mathit{Int} \mapsto 3,\ \ell_2\colon \mathsf{Ref}_{\mathsf{int}} \mapsto \ell_3]$. The heaplets $s_1$ and $s_2$ can be graphically presented as follows:

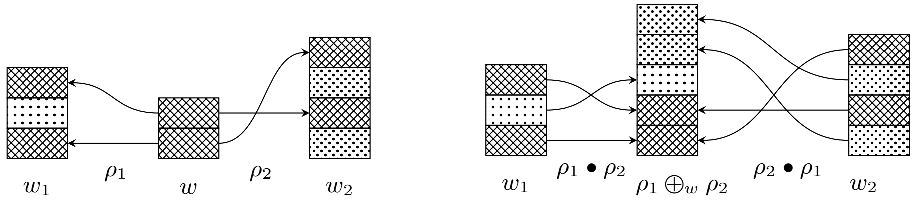

Fig. 3: Local independent coproduct

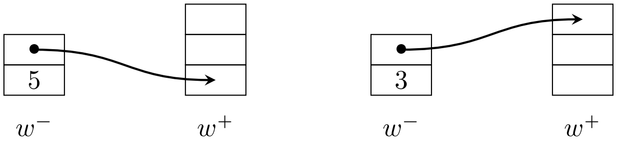

The category $\mathbf{W}$ supports (local) independent coproducts described in Section 2. These are constructed as follows. For $w, w' \in |\mathbf{C}|$, $w \oplus w' = w \cup \{\ell + n + 1 \colon S \mid (\ell, c) \in w'\}$ with $n$ being the largest index for which $w$ is defined on $\#^{-1}(n)$. This yields a strict monoidal structure $\oplus \colon \mathbf{W} \times \mathbf{W} \to \mathbf{W}$. Intuitively, $w_1 \oplus w_2$ is a canonical disjoint sum of $w_1$ and $w_2$, but note that $\oplus$ is not a coproduct in $\mathbf{W}$ (e.g. there is no $\nabla \colon 1 \oplus 1 \to 1$, for $\mathbf{W}$ only contains injections). For every $\rho \colon w_1 \to w_2$, there is a canonical complement $\rho^{\complement} \colon w_2 \ominus \rho \to w_2$ whose domain $w_2 \ominus \rho = w_2 \smallsetminus \mathsf{img}\,\rho$ consists of all such cells $(\ell \colon S) \in w_2$ that $\rho$ misses. Given two morphisms $\rho_1 \colon w \to w_1$ and $\rho_2 \colon w \to w_2$, we define the local independent coproduct $w_1 \oplus_w w_2$ as the layout consisting of the locations from $w$, and the ones from $w_1$ and $w_2$ which are neither in the image of $\rho_1$ nor in the image of $\rho_2$:

$$\rho_1 \oplus_w \rho_2 = w \oplus (w_1 \ominus \rho_1) \oplus (w_2 \ominus \rho_2).$$

There are morphisms $w_1 \xrightarrow{\rho_1 \bullet \rho_2} \rho_1 \oplus_w \rho_2$ and $w_2 \xrightarrow{\rho_2 \bullet \rho_1} \rho_1 \oplus_w \rho_2$ such that

$$\begin{array}{ccc} w & \xrightarrow{\rho_2} & w_2 \\ {\scriptstyle \rho_1}\downarrow & & \downarrow{\scriptstyle \rho_2 \bullet \rho_1} \\ w_1 & \xrightarrow{\rho_1 \bullet \rho_2} & \rho_1 \oplus_w \rho_2 \end{array}$$

Fig. 3 illustrates this definition with a concrete example.

**Initialization and hiding** Note that in the simple store model (Definition 3), $\mathcal{H}$ is equivalently a contravariant functor $H \colon \mathbf{W}^{\mathsf{op}} \to \mathbf{Set}$ with $Hw = \mathcal{V}^w$, hence $\mathcal{H}$ can be placed e.g. in $[\mathbf{W}^{\mathsf{op}}, \mathbf{Set}]$. In general, $\mathcal{H}$ is mix-variant, which calls for a more ingenious category where $H$ could be placed. Designing such category is indeed the key insight of [9]. Closely following this work, we introduce a category $\mathbf{E}$, whose objects are the same as those of $\mathbf{W}$, and the morphisms $\epsilon \in \mathbf{E}(w, w')$, called *initializations*, consist of an injection $\rho \colon w \to w'$ and a heaplet $\eta \in \mathcal{H}(w' \ominus \rho, w')$:

$$\mathbf{E}(w, w') = \sum\nolimits_{\rho\colon w \to w'} \mathcal{H}(w' \ominus \rho, w').$$

Recall that the morphism $\rho\colon w \to w'$ represents a move from a world with $w$ allocated memory cells a world with $w'$ allocated memory cells. A morphism of $\mathbf{E}$ is a morphism of $\mathbf{W}$ augmented with a heaplet part $\eta$, which provides the information how the newly allocated cells in $w' \ominus \rho$ are filled. The heap functor now can be viewed as a representable presheaf $H\colon \mathbf{E} \to \mathbf{Set}$ essentially because by definition, $Hw = \mathcal{H}(w, w) \cong \mathbf{E}(\emptyset, w)$. Let us agree to use the notation $\epsilon\colon w \rightsquigarrow w'$ for morphisms in $\mathbf{E}$ to avoid confusion with the morphisms in $\mathbf{W}$.

Like $\mathbf{W}$, $\mathbf{E}$ supports local independent coproducts, but remarkably $\mathbf{E}$ does not have vanilla independent coproducts, due to the fact that $\mathbf{E}$ does not have an initial object. That is, in turn, because defining an inital morphism would amount to defining canonical fresh values for newly allocated cells, but those need not exist. The local independent coproducts of $\mathbf{W}$ and $\mathbf{E}$ agree in the sense that we can *promote* an initialization $(\rho_2, \eta)\colon w \rightsquigarrow w_2$ along an injection $\rho_1\colon w \to w_1$ to obtain an initialization $\rho_1 \bullet (\rho_2, \eta)\colon w_1 \rightsquigarrow \rho_1 \oplus_{w_1} \rho_2$. This is accomplished by mapping the heaplet structure $\eta$ forward along $\rho_2 \bullet \rho_1\colon w_2 \to \rho_1 \oplus_w \rho_2$.

**Hiding monad** Recall that the local store is supposed to be insensitive to garbage collection. This is captured by identifying the stores that agree on their observable parts using the *hiding monad* $P$ defined on $[\mathbf{E}, \mathbf{Set}]$ as follows:

$$(PX)w = \int^{\rho\colon w \to w' \in w \downarrow \mathfrak{u}} Xw'. \tag{2}$$

Here, $\mathfrak{u}\colon \mathbf{E} \to \mathbf{W}$ is the obvious heaplet discarding functor $\mathfrak{u}(\rho, \eta) = \rho$. Intuitively, in (2), we view the locations of $w$ as public and the ones of $w' \ominus \rho$ as private. The integral sign denotes a *coend*, which in this case is just an ordinary colimit on $\mathbf{Set}$ and is computed as a quotient of $\sum_{\rho\colon w \to w' \in w \downarrow \mathfrak{u}} Xw'$ under the equivalence relation $\sim$ obtained as a symmetric-transitive closure of the relation

$$(\rho\colon w \to w_1, x \in Xw_1) \preceq (\mathfrak{u}\epsilon \circ \rho\colon w \to w_2, (X\epsilon)(x) \in Xw_2) \qquad (\epsilon\colon w_1 \rightsquigarrow w_2)$$

Note that $\preceq$ is a preorder. Moreover, it enjoys the following *diamond property.*

**Proposition 5.** *If* $(\rho, x) \preceq (\rho_1, x_1)$ *and* $(\rho, x) \preceq (\rho_2, x_2)$ *then* $(\rho_1, x_1) \preceq (\rho', x')$ *and* $(\rho_2, x_2) \preceq (\rho', x')$ *for a suitable* $(\rho', x')$.

*Hence* $(\rho_1, x_1) \sim (\rho_2, x_2)$ *iff* $(\rho_1, x_1) \preceq (\rho, x)$, $(\rho_2, x_2) \preceq (\rho, x)$ *for some* $(\rho, x)$.

**Example 6.** To illustrate the equivalence relation $\sim$ behind $P$, we revisit the setting of Example 4. Consider the following situations:

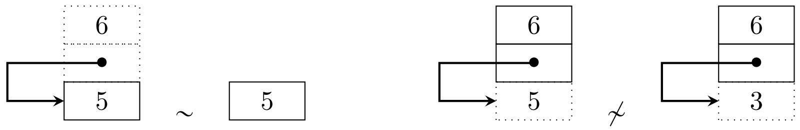

Here, the solid lines indicate public locations and the dotted lines indicate private locations. The left equivalence holds because the private locations are not reachable from the public ones by references (depicted as arrows). On the right, although the public parts are equal, the reachable cells of the private parts reveal the distinction, preventing the equivalence under $\sim$. Intuitively, hiding identifies those heaps that agree both on their public and reachable private part.

The covariant action of $PX$ (on $\mathbf{E}$) is defined via promotion of initializations:

$$\begin{aligned}(PX)(\epsilon\colon w_1 \rightsquigarrow w_2)(\rho\colon w_1 \to w_1', x \in Xw_1')_\sim \\ = (\mathfrak{u}\epsilon \bullet \rho\colon w_2 \to \rho \oplus_{w_1} \mathfrak{u}\epsilon, X(\rho \bullet \epsilon)(x))_\sim.\end{aligned}$$

Furthermore, there is a contravariant hiding operation (on $\mathbf{W}$) given by the canonical action of the coend: for $\rho\colon w \to w'$, we define $\mathsf{hide}_\rho\colon PXw' \to PXw$:

$$\mathsf{hide}_\rho(\rho'\colon w' \to w'', x \in Xw'')_\sim \;= (\rho' \circ \rho, x)_\sim \tag{3}$$

This allows us to regard $P$ both as a functor $[\mathbf{E}, \mathbf{Set}] \to [\mathbf{E}, \mathbf{Set}]$ and as a functor $[\mathbf{E}, \mathbf{Set}] \to [\mathbf{W}^{\mathsf{op}}, \mathbf{Set}]$.

**Full ground store monad** We now have all the necessary ingredients to obtain the full ground store monad $T$ on $[\mathbf{W}, \mathbf{Set}]$. This monad is assembled by composing the functors in Fig. 1 in the following way. First, observe that $(P(\text{-} \times H))^H$ is a standard (global) store monad transform of $P$ on $[\mathbf{E}, \mathbf{Set}]$. This monad is sandwiched between the adjunction $\mathfrak{u}_\star \dashv \mathfrak{u}^\star$ induced by $\mathfrak{u}$ (see Section 2). Since any monad itself resolves into an adjunction, sandwiching in it between an adjunction again yields a monad. In summary,

$$T = \Big([\mathbf{W}, \mathbf{Set}] \xrightarrow{\;\mathfrak{u}^\star\;} [\mathbf{E}, \mathbf{Set}] \xrightarrow{\;P(-\,\times\, H)^H\;} [\mathbf{E}, \mathbf{Set}] \xrightarrow{\;\mathfrak{u}_\star\;} [\mathbf{W}, \mathbf{Set}]\Big). \tag{4}$$

**Theorem 7.** *The monad $T$, defined by (4) is strong.*

*Proof.* The proof is a straightforward generalization of the proof in [9]. □

We can recover the monad previously developed by Plotkin and Power [16] by resorting to the simple store (Example 3).

**Theorem 8.** *Under the simple store model $T$ is isomorphic to the local store monad from [16]:*

$$(TX)w \cong \left(\int^{\rho\colon w \to w' \in w\downarrow \mathbf{W}} Xw' \times \mathcal{V}^{w'}\right)^{\mathcal{V}^w}.$$

Using (4), one obtains the requisite semantics to the language in Fig. 2 using the standard clauses of fine-grain call-by-value [11], except for the special clauses for **(put)**, **(get)** and **(new)**, which require special operations of the monad:

$$\begin{aligned}\mathsf{get}\colon\ & \mathfrak{u}^\star\underline{\mathsf{Ref}_S} \times H \to \mathfrak{u}^\star\underline{\mathsf{CType}(S)} \times H\\ \mathsf{put}\colon\ & (\mathfrak{u}^\star\underline{\mathsf{Ref}_S} \times \mathfrak{u}^\star\underline{\mathsf{CType}(S)}) \times H \to 1 \times H\\ \mathsf{new}\colon\ & \mathfrak{u}^\star(\underline{\mathsf{CType}(S)}^{\underline{\mathsf{Ref}_S}}) \times H \to P(\mathfrak{u}^\star\underline{\mathsf{Ref}_S} \times H)\end{aligned}$$

## 5 Intermezzo: BI-Hyperdoctrines and BI-Algebras

To be able to give a categorical notion of higher order logic over local store, following Biering et al [2], we aim to construct a *BI-hyperdoctrine*.

Note that algebraic structures, such as monoids and Heyting algebras can be straightforwardly internalized in any category with finite products, which gives rise to *internal monoids*, *internal Heyting algebras*, etc. The situation changes when considering non-algebraic properties. In particular, recall that a Heyting algebra $A$ is *complete* iff it has arbitrary joins, which are preserved by binary meets. The corresponding categorical notion is essentially obtained from spelling out generic definitions from internal category theory [6, B2] and is as follows.

**Definition 9 (Internally Complete Heyting Algebras).** An internal Heyting (Boolean) algebra $A$ in a finitely complete category $\mathbf{C}$ is *internally complete* if for every $f \in \mathbf{C}(I, J)$, there exist *indexed joins* $\bigvee_f : \mathbf{C}(I, A) \to \mathbf{C}(J, A)$, left order-adjoint to $(-) \circ f : \mathbf{C}(J, A) \to \mathbf{C}(I, A)$ such that for any pullback square on the left, the corresponding diagram on the right commutes (*Beck-Chevalley condition*):

$$
\begin{array}{ccc}
I & \xrightarrow{\;f\;} & J \\
{\scriptstyle g}\big\downarrow & \lrcorner & \big\downarrow{\scriptstyle h} \\
I' & \xrightarrow[\;f'\;]{} & J'
\end{array}
\qquad\qquad
\begin{array}{ccc}
\mathbf{C}(J, A) & \xrightarrow{\;(-)\circ f\;} & \mathbf{C}(I, A) \\
{\scriptstyle \bigvee_h}\big\downarrow & & \big\downarrow{\scriptstyle \bigvee_g} \\
\mathbf{C}(J', A) & \xrightarrow[\;(-)\circ f'\;]{} & \mathbf{C}(I', A)
\end{array}
$$

It follows generally that existence of indexed joins $\bigvee$ implies existence of indexed meets $\bigwedge$, which then satisfy dual conditions ([6, Corollary 2.4.8]).

**Remark 10 (Binary Joins/Meets).** The adjointness condition for indexed joins means precisely that $\bigvee_f \phi \leqslant \psi$ iff $\phi \leqslant \psi \circ f$ for every $\phi : I \to A$ and every $\psi : J \to A$. If $\mathbf{C}$ has binary coproducts, by taking $f = \nabla : X + X \to X$ we obtain that $\bigvee_\nabla \phi \leqslant \psi$ iff $\phi \leqslant [\psi, \psi]$ iff $\phi \circ \mathsf{inl} \leqslant \psi$ and $\phi \circ \mathsf{inr} \leqslant \psi$. This characterizes $\bigvee_\nabla [\phi_1, \phi_2] : X \to A$ as the binary join of $\phi_1, \phi_2 : X \to A$. Binary meets are characterized analogously.

**Definition 11 ((First Order) (BI-)Hyperdoctrine).** Let $\mathbf{C}$ be a category with finite products. A *first order hyperdoctrine over* $\mathbf{C}$ is a functor $S : \mathbf{C}^{\mathsf{op}} \to$ **Poset** with the following properties:

1. given $X \in |\mathbf{C}|$, $SX$ is a Heyting algebra;
2. given $f \in \mathbf{C}(X, Y)$, $Sf : SY \to SX$ is a Heyting algebra morphism;
3. for any product projection $\mathsf{fst} : X \times Y \to X$, there are $(\exists Y)_X : S(X \times Y) \to SX$ and $(\forall Y)_X : S(X \times Y) \to SX$, which are respective left and right order-adjoints of $S\,\mathsf{fst} : S(X \times Y) \to SX$, naturally in $X$;
4. for every $X \in |\mathbf{C}|$, there is $=_X \in S(X \times X)$ such that for all $\phi \in S(X \times X)$, $\top \leqslant (S\langle \mathsf{id}_X, \mathsf{id}_X\rangle)(\phi)$ iff $=_X \leqslant \phi$.

If additionally

$$\frac{\Gamma \vdash_{\mathsf{v}} v\colon A \quad \Gamma \vdash \phi\colon \mathsf{P}A}{\Gamma \vdash \phi(v)\colon \mathrm{prop}} \qquad \frac{\Gamma, x\colon A \vdash \phi\colon \mathrm{prop}}{\Gamma \vdash x.\,\phi\colon \mathsf{P}A} \qquad \frac{\Gamma \vdash_{\mathsf{v}} \ell\colon \mathsf{Ref}_S \quad \Gamma \vdash_{\mathsf{v}} v\colon \mathsf{CType}(S)}{\Gamma \vdash \ell \hookrightarrow v\colon \mathrm{prop}}$$

$$\frac{\Gamma \vdash \phi\colon \mathsf{P}A}{\Gamma \vdash Q\,\phi\colon \mathrm{prop}}\ (Q \in \{\forall, \exists\}) \qquad \frac{\Gamma \vdash_{\mathsf{v}} v\colon A \qquad \Gamma \vdash_{\mathsf{v}} w\colon A}{\Gamma \vdash v = w\colon \mathrm{prop}}$$

$$\frac{}{\Gamma \vdash c\colon \mathrm{prop}}\ (c \in \{\top, \bot\}) \qquad \frac{\Gamma \vdash \phi\colon \mathrm{prop} \qquad \Gamma \vdash \psi\colon \mathrm{prop}}{\Gamma \vdash \phi\,\$\,\psi\colon \mathrm{prop}}\ (\$ \in \{\wedge, \vee, \Rightarrow, \star, -\!\!\star\})$$

Fig. 4: Term formation rules for the higher order separation logic.

5. given $X \in |\mathbf{C}|$, $SX$ is a *BI-algebra*, i.e. a commutative monoid equipped with a right order-adjoint to multiplication;
6. given $f \in \mathbf{C}(X, Y)$, $Sf\colon SY \to SX$ is a BI-algebra morphism, then $S$ is called a *first order BI-hyperdoctrine.*

In a *(higher order) hyperdoctrine*, $\mathbf{C}$ is additionally required to be Cartesian closed and every $SX$ is required to be poset-isomorphic to $\mathbf{C}(X, A)$ for a suitable internal Heyting algebra $A \in |\mathbf{C}|$ naturally in $X$. Such a hyperdoctrine is a *BI-hyperdoctrine* if moreover $A$ is an internal BI-algebra.

**Proposition 12.** *Every internally complete Heyting algebra $A$ in a Cartesian closed category $\mathbf{C}$ with finite limits gives rise to a canonical hyperdoctrine $\mathbf{C}(-, A)$: for every $X$, $\mathbf{C}(X, A)$ is a poset under $f \leqslant g$ iff $f \wedge g = f$.*

*Proof.* Clearly, every $\mathbf{C}(X, A)$ is a Heyting algebra and every $\mathbf{C}(f, A)$ is a Heyting algebra morphism. The quantifies are defined mutually dually as follows:

$$(\exists Y)_X(\phi\colon X \times Y \to A) = \bigvee\nolimits_{\mathsf{fst}\colon X\times Y \to X} \phi,$$
$$(\forall Y)_X(\phi\colon X \times Y \to A) = \bigwedge\nolimits_{\mathsf{fst}\colon X\times Y \to X} \phi.$$

Naturality in $X$ follows from the corresponding Beck-Chevalley conditions.

Finally, internal equality $=_X\colon X \times X \to A$ is defined as $\bigvee_{\langle \mathsf{id}_X, \mathsf{id}_X\rangle} \top$. $\square$

A standard way to obtain an (internally) complete BI-algebra is to resort to ordered partial commutative monoids [18].

**Definition 13 (Ordered PCM [18]).** An *ordered partial commutative monoid (pcm)* is a tuple $(\mathcal{M}, \mathcal{E}, \cdot, \leqslant)$ where $\mathcal{M}$ is a set, $\mathcal{E} \subseteq \mathcal{M}$ is a set of *units*, *multiplication* $\cdot$ is a partial binary operation on $\mathcal{M}$, and $\leqslant$ is a preorder on $\mathcal{M}$, satisfying an number of axioms (see [18] for details).

We note that using general recipes [3], for every internal ordered pcm $M$ in a topos $\mathbf{C}$ with subobject classifier $\Omega$, $\mathbf{C}(- \times M, \Omega)$ forms a BI-hyperdoctrine, on particular, if $\mathbf{C} = \mathbf{Set}$ then $\mathbf{Set}(- \times M, 2)$ is a BI-hyperdoctrine.

## 6 A Higher Order Logic for Full Ground Store

We proceed to develop a local version of separation logic using semantic principles explored in the previous sections. That is, we seek an interpretation for the language in Fig. 4 in the category $[\mathbf{W}, \mathbf{Set}]$ over the type system (1), extended with *predicate types* $\mathsf{P}A$. The judgements $\Gamma \vdash \phi$: prop type formulas depending on a variable context $\Gamma$. Additionally, we have judgements of the form $\Gamma \vdash \phi\colon \mathsf{P}A$ for *predicates in context.* Both kinds of judgements are mutually convertible using the standard application-abstraction routine. Note that expressions for quantifiers $\exists x.\,\phi$ are thus obtained in two steps: by forming a predicate $x.\,\phi$, and subsequently applying $\exists$. Apart from the standard logical connectives, we postulate *separating conjunction* $\star$ and *separating implication* $-\!\!\star$.

Our goal is to build a BI-hyperdoctrine, using the recipes, summarized in the previous section. That is, we construct a certain internal BI-algebra $\Theta$ in $[\mathbf{W}, \mathbf{Set}]$, and subsequently conclude that $[-, \Theta]$ is a BI-hyperdoctrine in question. In what follows, most of the effort is invested into constructing an internally complete Boolean algebra $\check{\mathcal{P}} \circ (\hat{P}\hat{H})$ (hence $[-, \check{\mathcal{P}} \circ (\hat{P}\hat{H})]$ is a hyperdoctrine), from which $\Theta$ is carved out as a subfunctor, identified by an upward closure condition. Here, $\check{\mathcal{P}}$ is a contravariant powerset functor, and $\hat{P}$ and $\hat{H}$ are certain modifications of the hiding and the heap functors from Section 4. As we shall see, the move from $\check{\mathcal{P}} \circ (\hat{P}\hat{H})$ to $\Theta$ remedies the problem of the former that the natural separation conjunction operator $\star$ on it does not have unit (Remark 19).

In order to model resource separation, we must identify a domain of logical assertions over partial heaps, i.e. heaplets, instead of total heaps. We thus need to derive a unary (covariant) heaplet functor from the binary, mix-variant one $\mathcal{H}$ used before. We must still cope not only with heaplets, but with partially hidden heaplets, to model information hiding. A seemingly natural candidate functor for hidden heaplets is the composition

$$P\big(\mathbf{E} \xrightarrow{\sum_{w \subseteq -} \mathcal{H}(w,-)} \mathbf{Set}\big)\colon \mathbf{W}^{\mathsf{op}} \to \mathbf{Set}.$$

One problem of this definition is that the equivalence relation $\sim$ underlying the construction of $P$ (2) is too fine. Consider, for example, $e_w = (\emptyset \subseteq w, \star) \in \sum_{w' \subseteq w} \mathcal{H}(w', w)$. Then $(\mathsf{id}\colon w \to w, e_w) \not\sim (\mathsf{inl}\colon w \to w \oplus \{\star\colon 1\}, e_{w \oplus \{\star\colon 1\}})$, i.e. two hidden heaplets would not be equivalent if one extends the other by an inaccessible hidden cell. In order to arrive at a more reasonable model of logical assertions, we modify the previous model by replacing the category of initializations $\mathbf{E}$ is a category $\hat{\mathbf{E}}$ of *partial initializations.* This will induce a hiding monad $\hat{P}$ over $[\hat{\mathbf{E}}, \mathbf{Set}]$ using exactly the same formula (2) as for $P$.

A partial initialization is a pair $(\rho, \eta)$ with $\rho \in \mathbf{W}(w_1^-, w_2^+)$ and $\eta \in \sum_{w^- \subseteq w_2^+ \ominus \rho} \mathcal{H}(w^-, w_2^+)$. Let $\hat{\mathbf{E}}$ be the category of heap layouts and partial initializations. Analogously to $\mathfrak{u}$, there is an obvious partial-heap-forgetting functor $\hat{\mathfrak{u}}\colon \hat{\mathbf{E}} \to \mathbf{W}$. Let $\hat{H}\colon \hat{\mathbf{E}} \to \mathbf{Set}$ be the following *heaplet functor*:

$$\hat{H}w = \sum\nolimits_{w' \subseteq w} \mathcal{H}(w', w).$$

Given a partial initialization $\epsilon = (\rho\colon w \to w', (w'' \subseteq w' \ominus \rho, \eta \in \mathcal{H}(w'', w')))\colon w \rightsquigarrow w'$, $\hat{H}\epsilon\colon \hat{H}w \to \hat{H}w'$ extends a given heaplet over $w$ to a heaplet over $w'$ via $\eta$:

$$(\hat{H}\epsilon)(w_1 \subseteq w, \eta' \in \mathcal{H}(w_1, w)) = (\rho[w_1] \cup w'' \subseteq w', \eta'')$$

where $\eta'' \in \mathcal{H}(\rho[w_1] \cup w'' \subseteq w', w')$ is as follows

$$\mathsf{pr}_{\rho(\ell\colon S)}\,\eta'' = \mathsf{range}(S)(\rho)(\mathsf{pr}_{(\ell\colon S)}\,\eta') \qquad ((\ell\colon S) \in w_1)$$
$$\mathsf{pr}_{(\ell\colon S)}\,\eta'' = \mathsf{pr}_{(\ell\colon S)}\,\eta \qquad ((\ell\colon S) \in w'')$$

With $\hat{\mathbf{E}}$ and $\hat{H}$ as above instead of $\mathbf{E}$ and $H$, the framework described in Section 4 transforms coherently.

**Remark 14.** Let us fix a fresh symbol ⊠, and note that

$$\hat{H}w = \sum\nolimits_{w' \subseteq w} \prod\nolimits_{(\ell\colon S) \in w'} \mathsf{range}(S)(w) \cong \prod\nolimits_{(\ell\colon S) \in w} (\mathsf{range}(S)(w) \uplus \{\boxtimes\}),$$

meaning that the passage from $\mathbf{E}$, $H$ and $P$ to $\hat{\mathbf{E}}$, $\hat{H}$ and $\hat{P}$ is equivalent to extending the $\mathsf{range}$ function with designated values ⊠ for *inaccessible locations*. We prefer to think of ⊠ this way and not as a content of *dangling pointers*, to emphasize that we deal with a *reasoning phenomenon* and not with a *programming phenomenon*, for our programs neither create nor process dangling pointers.

For the next proposition we need the following concrete description of the set $\hat{\mathfrak{u}}_\star(2^X)w$ as the end $\int_{\rho\colon w \to w' \in w \downarrow \hat{\mathfrak{u}}} \mathbf{Set}(Xw', 2)$: this set is a space of dependent functions $\phi$ sending every injection $\rho\colon w \to w'$ to a corresponding subset of $Xw'$, and satisfying the constraint: $x \in \phi(\rho)$ iff $(X\,\epsilon)(x) \in \phi(\hat{\mathfrak{u}}\,\epsilon \circ \rho)$ for every $\epsilon\colon w' \rightsquigarrow w''$.

**Proposition 15.** *The following diagram commutes up to isomorphism:*

$$\begin{array}{ccc}
[\hat{\mathbf{E}}, \mathbf{Set}] & \xrightarrow{\;2^{(-)}\;} & [\hat{\mathbf{E}}, \mathbf{Set}]^{\mathsf{op}} \\
\downarrow{\scriptstyle \hat{P}} & & \downarrow{\scriptstyle \hat{\mathfrak{u}}_\star} \\
[\mathbf{W}, \mathbf{Set}^{\mathsf{op}}]^{\mathsf{op}} & \xrightarrow{\;\check{\mathcal{P}} \circ (-)\;} & [\mathbf{W}, \mathbf{Set}]^{\mathsf{op}}
\end{array}$$

*(using the fact that* $[\mathbf{W}, \mathbf{Set}^{\mathsf{op}}]^{\mathsf{op}} \cong [\mathbf{W}^{\mathsf{op}}, \mathbf{Set}]$*) where* $\check{\mathcal{P}}$ *is the contravariant powerset functor* $\check{\mathcal{P}}\colon \mathbf{Set}^{\mathsf{op}} \to \mathbf{Set}$ *and for every* $X\colon \hat{\mathbf{E}} \to \mathbf{Set}$ *the relevant isomorphism* $\Phi_w\colon \hat{\mathfrak{u}}_\star(2^X)w \cong \check{\mathcal{P}}(\hat{P}Xw)$ *is as follows:*

$$(\rho\colon w \to w', x \in Xw')_\sim \in \Phi_w(\phi \in \hat{\mathfrak{u}}_\star(2^X)w) \iff x \in \phi(\rho). \tag{5}$$

Let us clarify the significance of Proposition 15. The exponential $2^{\hat{H}}$ in $[\hat{\mathbf{E}}, \mathbf{Set}]$ can be thought of as a carrier of Boolean predicates over $\hat{H}$, and as we see next those form an internally complete Boolean algebra, which is carried from $[\hat{\mathbf{E}}, \mathbf{Set}]$ to $[\mathbf{W}, \mathbf{Set}]$ by $\hat{\mathfrak{u}}_\star$. The alternative route via $\hat{P}$ and $\check{\mathcal{P}}$ induces a Boolean algebra of predicates over hidden heaplets $\hat{P}\hat{H}$ directly in $[\mathbf{W}, \mathbf{Set}]$. The equivalence established in Proposition 15 witnesses agreement of these two structures.

**Theorem 16.** *For every $X\colon \hat{\mathbf{E}} \to \mathbf{Set}$, $\check{\mathcal{P}} \circ (\hat{P}X)$ is an internally complete Boolean algebra in $[\mathbf{W}, \mathbf{Set}]$ under*

$$\begin{aligned}\Big(\bigvee\nolimits_f \phi\colon I \to \check{\mathcal{P}} \circ (\hat{P}X)\Big)_w (j \in Jw)&\\ = \{(\rho\colon w \to w', x \in Xw')_\sim \mid \exists\, \epsilon\colon w' \rightsquigarrow w'', \exists i \in Iw''.&\\ f_{w''}(i) = J(\hat{\mathfrak{u}}\,\epsilon \circ \rho)(j) \wedge (\mathsf{id}_{w''}, (X\,\epsilon)(x))_\sim \in \phi_{w''}(i)\},&\\ \Big(\bigwedge\nolimits_f \phi\colon I \to \check{\mathcal{P}} \circ (\hat{P}X)\Big)_w (j \in Jw)&\\ = \{(\rho\colon w \to w', x \in Xw')_\sim \mid \forall\, \epsilon\colon w' \rightsquigarrow w'', \forall i \in Iw''.&\\ f_{w''}(i) = J(\hat{\mathfrak{u}}\,\epsilon \circ \rho)(j) \Rightarrow (\mathsf{id}_{w''}, (X\,\epsilon)(x))_\sim \in \phi_{w''}(i)\}.&\end{aligned}$$

*for every $f\colon I \to J$, and the corresponding Boolean algebra operations are computed as set-theoretic unions, intersections and complements.*

By Theorem 16, we obtain a hyperdoctrine $[-, \check{\mathcal{P}} \circ (\hat{P}\hat{H})]$, which provides us with a model of (classical) higher order logic in $[\mathbf{W}, \mathbf{Set}]$. In particular, this allows us to interpret the language from Fig. 4 over $[\mathbf{W}, \mathbf{Set}]$ excluding the separation logic constructs, in such a way that

$$[\![\Gamma \vdash \phi\colon \mathrm{prop}]\!]\colon \underline{\Gamma} \to \check{\mathcal{P}} \circ (\hat{P}\hat{H}), \qquad [\![\Gamma \vdash \phi\colon \mathsf{P}A]\!]\colon \underline{\Gamma} \times \underline{A} \to \check{\mathcal{P}} \circ (\hat{P}\hat{H})$$

where $\underline{\Gamma} = \underline{A_1} \times \ldots \times \underline{A_n}$ for $\Gamma = (x_1\colon A_1, \ldots, x_n\colon A_n)$ where, additionally to the standard clauses, $\underline{\mathsf{P}A} = \check{\mathcal{P}} \circ \hat{P}(\mathfrak{u}^\star \underline{A} \times \hat{H})$. The latter interpretation of predicate types $\mathsf{P}A$ is justified by the natural isomorphism:

$$(\check{\mathcal{P}} \circ (\hat{P}\hat{H}))^X \cong (\hat{\mathfrak{u}}_\star(2^{\hat{H}}))^X \cong \hat{\mathfrak{u}}_\star((2^{\hat{H}})^{\hat{\mathfrak{u}}^\star X}) \cong \check{\mathcal{P}} \circ (\hat{P}(\hat{\mathfrak{u}}^\star X \times \hat{H})).$$

Here, the first and the last transitions are by $\Phi$ from Proposition 15 and the middle one is due to the fact that clearly both $(\hat{\mathfrak{u}}_\star(-))^X \vdash \hat{\mathfrak{u}}^\star(X \times (-))$ and $\hat{\mathfrak{u}}_\star((-)^{\hat{\mathfrak{u}}^\star X}) \vdash \hat{\mathfrak{u}}^\star(X \times (-))$.

Since every set $\hat{H}w$ models a heaplet in the standard sense [18], we can equip $\hat{H}w$ with a standard pointer model structure.

**Proposition 17.** *For every $w \in |\mathbf{W}|$, $(\hat{H}w, \{(\emptyset \subseteq w, \star)\}, \cdot, \leqslant)$ is an ordered pcm where for every $w \in |\mathbf{W}|$, $\hat{H}w$ is partially ordered as follows:*

$$(w_1 \subseteq w, \mathcal{H}(w_1 \subseteq w_2, w)\eta \in \mathcal{H}(w_1, w)) \leqslant (w_2 \subseteq w,\ \eta \in \mathcal{H}(w_2, w)) \quad (w_1 \subseteq w_2)$$

*and for $w_1 \subseteq w$, $w_2 \subseteq w$ and $\eta_1 \in \mathcal{H}(w_1, w)$, $\eta_2 \in \mathcal{H}(w_2, w)$, $(w_1 \subseteq w, \eta_1) \cdot (w_2 \subseteq w, \eta_2)$ equals $(w_1 \cup w_2, \eta_1 \cup \eta_2)$ if $w_1 \cap w_2 = \emptyset$, and otherwise undefined.*

As indicated in Section 5, we automatically obtain a BI-algebra structure over the set of all subsets of $\hat{H}w$. The same strategy does not apply to $\hat{P}\hat{H}w$, roughly because we cannot predict mutual arrangement of hidden partitions of two heaplets wrt to each other, for we do not have a global reference space for pointers as contrasted to the standard separation logic setting. We thus define a separating conjunction operator directly on every $\check{\mathcal{P}}(\hat{P}\hat{H}w)$ as follows:

$$\begin{aligned}\phi \star_w \psi = \{(\rho\colon w \to w', (w_1 \uplus w_2 \subseteq w', \eta \in \mathcal{H}(w_1 \uplus w_2, w')))_\sim \mid \\ (\rho, (w_1 \subseteq w', \mathcal{H}(w_1 \subseteq w_1 \uplus w_2, w')\eta))_\sim \in \phi, \\ (\rho, (w_2 \subseteq w', \mathcal{H}(w_2 \subseteq w_1 \uplus w_2, w')\eta))_\sim \in \psi\}.\end{aligned}$$

**Lemma 18.** *The operator $\star_w$ on $\check{\mathcal{P}}(\hat{P}\hat{H}w)$ satisfies the following properties.*

1. *$\star_w$ is natural in $w$.*
2. *$\star_w$ is associative and commutative.*
3. *$(\rho\colon w \to w', (w'' \subseteq w', \eta \in \mathcal{H}(w'', w')))_\sim \in \phi \star_w \psi$ if and only if there exist $w_1$, $w_2$ such that $w_1 \uplus w_2 = w''$, $(\rho, (w_1 \subseteq w', \mathcal{H}(w_1 \subseteq w'', w')\eta))_\sim \in \phi$ and $(\rho, (w_2 \subseteq w', \mathcal{H}(w_2 \subseteq w'', w')\eta))_\sim \in \psi$.*

Property (3) specifically tells us that any representative of an equivalence class contained in a separating conjunction can be split in such a way that the respective pieces belong to the arguments of the separating conjunction.

**Remark 19.** The only candidate for the unit of the separating conjunction $\star_w$ would be the emptiness predicate $\mathsf{empty}_w\colon 1 \to \check{\mathcal{P}}(\hat{P}\hat{H}w)$, identifying precisely the empty heaplets. However, $\mathsf{empty}_w$ is not natural in $w$. In fact, it follows by Yoneda lemma that there are exactly two natural transformations $1 \to \check{\mathcal{P}} \circ (\hat{P}\hat{H})$, which are the total truth and the total false, none of which is a unit for $\star_w$.

Remark 19 provides a formal argument why we cannot interpret classical separation logic over $\check{\mathcal{P}} \circ (\hat{P}\hat{H})$. We thus proceed to identify for every $w$ a subset of $\check{\mathcal{P}}(\hat{P}\hat{H}w)$, for which the total truth predicate becomes the unit of the separating conjunction. Concretely, let $\Theta$ be the subfunctor of $\check{\mathcal{P}} \circ (\hat{P}\hat{H})$ identified by the following *upward closure condition:* $\phi \in \Theta w$ if

$$(\rho, \eta)_\sim \in \phi,\ \eta \leqslant \eta' \qquad \text{imply} \qquad (\rho, \eta')_\sim \in \phi.$$

**Lemma 20.** *$\Theta$ is an internal complete sublattice of $\check{\mathcal{P}} \circ (\hat{P}\hat{H})$, i.e. the inclusion $\iota\colon \Theta \hookrightarrow \check{\mathcal{P}} \circ (\hat{P}\hat{H})$ preserves all meets and all joins. This canonically equips $\Theta$ with an internally complete Heyting algebra structure.*

*Proof (Sketch).* The key idea is to establish a retraction $(\iota, \mathsf{cl})$ with $\mathsf{cl} \circ \iota = \mathsf{id}$. The requisite structure is then transferred from $\check{\mathcal{P}} \circ (\hat{P}\hat{H})$ to $\Theta$ along it. The Heyting implication for $\Theta$ is obtained using the standard formula $(\phi \Rightarrow \psi) = \bigvee\{\xi \mid \phi \wedge \xi \leqslant \psi\}$ interpreted in the internal language. $\square$

**Lemma 21.** *Separating conjunction preserves upward closure: for $\phi, \psi \in \Theta w$, $\phi \star_w \psi = \mathsf{cl}_w(\phi \star_w \psi)$.*

**Lemma 22.** *$\Theta$ is a BI-algebra: $\star_w$ is obtained by restriction from $\check{\mathcal{P}}(\hat{P}\hat{H}w)$ by Lemma 21, $\hat{P}\hat{H}w$ is the unit for it and*

$$\begin{aligned}\phi \mathrel{-\!\!\star}_w \psi = \{(\rho, \eta)_\sim \in \Theta w \mid \forall \rho'\colon w \to w', \eta_1, \eta_2 \in \hat{H}w', \eta_1 \cdot \eta_2 \text{ defined} \wedge \\ (\rho, \eta) \sim (\rho', \eta_1) \wedge (\rho', \eta_2)_\sim \in \phi \Rightarrow (\rho', \eta_1 \cdot \eta_2)_\sim \in \psi\}.\end{aligned}$$

- $s, \rho, \eta \models \top$
- $s, \rho, \eta \models \phi \wedge \psi$ if $s, \rho, \eta \models \phi$ and $s, \rho, \eta \models \psi$
- $s, \rho, \eta \models \phi \vee \psi$ if $s, \rho, \eta \models \phi$ or $s, \rho, \eta \models \psi$
- $s, \rho, \eta \models \phi \Rightarrow \psi$ if for all $(\rho, \eta) \sim (\rho', \eta')$ and $\eta' \leqslant \eta''$,
  $s, \rho', \eta'' \models \phi$ implies $s, \rho', \eta'' \models \psi$
- $s, \rho, \eta \models \phi(v)$ if $s, \rho, ((\llbracket \Gamma \vdash_{\mathsf{v}} v\colon A \rrbracket_{w'} \circ \underline{\Gamma}\rho)s, \eta) \models \phi$
- $s, \rho, (a, \eta) \models x.\,\phi$ if $a = (X\rho)b$ and $(s, b), \rho, \eta \models \phi$
- $s, \rho, \eta \models \ell \hookrightarrow v$ if $\eta = (w'' \subseteq w', \delta \in \mathcal{H}(w'', w'))$ and
  $\delta(r\colon S) = (\llbracket \Gamma \vdash_{\mathsf{v}} v\colon \mathsf{CType}(S) \rrbracket_{w'} \circ \underline{\Gamma}\rho)s$
  where $(\llbracket \Gamma \vdash_{\mathsf{v}} \ell\colon \mathsf{Ref}_S \rrbracket_{w'} \circ \underline{\Gamma}\rho)s = (r\colon S) \in w''$
- $s, \rho, \eta \models v = u$ if $(\llbracket \Gamma \vdash_{\mathsf{v}} v\colon A \rrbracket_{w''} \circ \underline{\Gamma}\rho' \circ \underline{\Gamma}\rho)(s) = (\llbracket \Gamma \vdash_{\mathsf{v}} u\colon A \rrbracket_{w''} \circ \underline{\Gamma}\rho' \circ \underline{\Gamma}\rho)(s)$
  for some $\rho'\colon w' \to w''$
- $s, \rho, \eta \models \phi \star \psi$ if for suitable $w_1$, $w_2$, $\eta \in \mathcal{H}(w_1 \uplus w_2, w')$,
  $s, \rho, (w_1 \subseteq w', \mathcal{H}(w_1 \subseteq w_1 \uplus w_2, w')\eta) \models \phi$ and
  $s, \rho, (w_2 \subseteq w', \mathcal{H}(w_2 \subseteq w_1 \uplus w_2, w')\eta) \models \psi$
- $s, \rho, \eta \models \phi \mathrel{-\!\!\star} \psi$ if for all $(\rho', \eta_1) \sim (\rho, \eta)$ and for all $\eta_2$ such that $\eta_1 \cdot \eta_2$ is defined,
  $s, \rho', \eta_2 \models \phi$ implies $s, \rho', \eta_1 \cdot \eta_2 \models \psi$
- $s, \rho, \eta \models \exists \phi$ if $\underline{\Gamma}(\hat{\mathfrak{u}}\epsilon \circ \rho)s, \mathsf{id}_{w''}, (a, \hat{H}\epsilon \circ \eta) \models \phi$ for some $\epsilon\colon w' \rightsquigarrow w''$, $a \in \underline{A}w''$
- $s, \rho, \eta \models \forall \phi$ if $\underline{\Gamma}(\hat{\mathfrak{u}}\epsilon \circ \rho)s, \mathsf{id}_{w''}, (a, \hat{H}\epsilon \circ \eta) \models \phi$ for all $\epsilon\colon w' \rightsquigarrow w''$, $a \in \underline{A}w''$

Fig. 5: Semantics of the logic.

*Proof.* In view of Lemma 20, we are left to show that the given operations are natural and that $\Theta$ is an internal BI-algebra w.r.t. them. Since BI-algebras form a variety [5], it suffices to show that each $\Theta w$ is a BI-algebra. By Lemma 18 (ii), it suffices to show that every $(-) \star_w \phi$ preserves arbitrary joins, for then we can use the standard formula to calculate $\phi \mathrel{-\!\!\star}_w \psi$, which happens to be natural in $w$:

$$\phi \mathrel{-\!\!\star}_w \psi = \bigcup \{\xi \mid \phi \star_w \xi \leqslant \psi\}.$$

By unfolding the right-hand side, we obtain the expression for $\mathrel{-\!\!\star}_w$ figuring in the statement of the lemma. □

**Theorem 23.** *$\Theta$ is an internally complete Heyting BI-algebra, hence $[-, \Theta]$ is a BI-hyperdoctrine.*

*Proof.* Follows from Lemmas 20 and 22. □

This now provides us with a complete semantics of the language in Fig. 4 with $\llbracket \Gamma \vdash \phi\colon \text{prop} \rrbracket\colon \underline{\Gamma} \to \Theta$ and $\llbracket \Gamma \vdash \phi\colon \mathsf{P}A \rrbracket\colon \underline{\Gamma} \to \underline{\mathsf{P}A}$ where $\underline{\mathsf{P}A}$ is the upward closed subfunctor of $\check{\mathcal{P}} \circ (\hat{P}(\hat{\mathfrak{u}}\underline{A} \times \hat{H}))$, with upward closure only on the $\hat{H}$-part, which is isomorphic to $\Theta^{\underline{A}}$. The resulting semantics is defined in Fig. 5 where we write $s, \rho, \eta \models \phi$ for $(\rho, \eta)_\sim \in [\![\Gamma \vdash \phi\colon \mathrm{prop}]\!](s)$ and $s, \rho, (a, \eta) \models \phi$ for $(\rho, (a, \eta))_\sim \in [\![\Gamma \vdash \phi\colon \mathsf{P}A]\!](s)$. The following properties [4] are then automatic.

**Proposition 24.** – (Monotonicity) *If* $s, \rho, \eta \models \phi$ *and* $\eta \leqslant \eta'$ *then* $s, \rho, \eta' \models \phi$.
– (Shrinkage) *If* $s, \rho, \eta \models \phi$, $\eta' \leqslant \eta$ *and* $\eta'$ *contains all cells reachable from* $s$ *and* $w$ *then* $s, \rho, \eta' \models \phi$.

## 7 Examples

Let us illustrate subtle features of our semantics by some examples.

**Example 25.** Consider the formula $\exists \ell\colon \mathsf{Ref}_{\mathsf{Int}}.\, \ell \hookrightarrow 5$ from the introduction in the empty context –. Then $-, \rho, \eta \models \exists \ell.\, \ell \hookrightarrow 5$ iff for some $\epsilon\colon w' \rightsquigarrow w''$, and some $x \in \underline{\mathsf{Ref}_{\mathsf{Int}}} w''$, $x, \mathsf{id}_{w''}, (\hat{H}\epsilon)\eta \models \ell' \hookrightarrow 5$. The latter is true iff $\mathsf{pr}_x((\hat{H}\epsilon)\eta) = 5$. Note that $w'$ may not contain $\ell$ and it is always possible to choose $\epsilon$ so that $w''$ contains $\ell$ and $\mathsf{pr}_x((\hat{H}\epsilon)\eta) = 5$. Hence, the original formula is always valid.

**Example 26.** The clauses in Fig. 5 are very similar to the standard Kripke semantics of intuitionistic logic. Note however, that the clause for implication strikingly differs from the expected one

– $s, \rho, \eta \models \phi \Rightarrow \psi$ if for all $\eta \leqslant \eta'$, $s, \rho, \eta' \models \phi$ implies $s, \rho, \eta' \models \psi$, though. The latter is indeed not validated by our semantics, as witnessed by the following example. Consider the following formulas $\phi$ and $\psi$ respectively:

$$\ell\colon \mathsf{Ref}_{\mathsf{Ref}_{Int}} \vdash \exists \ell'.\, \exists x.\, \ell \hookrightarrow \ell' \wedge \ell' \hookrightarrow x\colon \mathrm{prop} \tag{6}$$

$$\ell\colon \mathsf{Ref}_{\mathsf{Ref}_{Int}} \vdash \exists \ell'.\, \ell \hookrightarrow \ell' \wedge \ell' \hookrightarrow 6\colon \mathrm{prop} \tag{7}$$

The first formula is valid over heaplets, in which $\ell$ refers to a reference to some integer, while the second one is only valid over heaplets, in which $\ell$ refers to a reference to 6. Any $\eta' \geqslant \eta = (\mathsf{id}_w, (\{\ell''\} \subseteq \{\ell, \ell''\}, [\ell'' \mapsto 6]))$ satisfies both (6) and (7) or none of them. However, the implication $\phi \Rightarrow \psi$ still is not valid over $\eta$ in our semantics, for

$$\begin{aligned} \eta &\sim (w \hookrightarrow w \oplus (\ell'\colon \mathit{Int}), (\{\ell', \ell''\} \subseteq \{\ell, \ell', \ell''\}, [\ell' \mapsto 5, \ell'' \mapsto 6])) \\ &\leqslant (w \hookrightarrow w \oplus (\ell'\colon \mathit{Int}), (\{\ell, \ell', \ell''\} \subseteq \{\ell, \ell', \ell''\}, [\ell \mapsto \ell', \ell' \mapsto 5, \ell'' \mapsto 6])) \end{aligned}$$

and the latter heaplet validates $\phi$ but not $\psi$.

**Example 27.** Least $\mu$ and greatest $\nu$ fixpoints can be encoded in higher order logic [2]. As an example, consider

$$\mathit{isList} = \mu\gamma.\, \ell.\ \ell \hookrightarrow \mathit{null} \vee \exists \ell', x.\, \ell \hookrightarrow (x, \ell') \star \gamma(\ell'),$$

which specifies the fact that $\ell$ is a pointer to a head of a list (eliding coproduct injections in $\mathsf{inl}\, \mathit{null}$ and $\mathsf{inr}(x, \ell')$). By definition, *isList* satisfies the following recursive equation:

$$\mathit{isList}(\ell) = \ell \hookrightarrow \mathit{null} \vee \exists \ell', x.\, \ell \hookrightarrow (x, \ell') \star \mathit{isList}(\ell')$$

Let us expand the semantics of the right hand side. We have

$$\begin{aligned}
&[\![\ell\colon \mathsf{Ref}_{\mathsf{list}}, \mathit{isList}\colon \mathsf{P}(\mathsf{Ref}_{\mathsf{list}}) \vdash l \hookrightarrow \mathit{null} \vee \exists \ell', x.\, \ell \hookrightarrow (x,\ell') \star \mathit{isList}(\ell')]\!]_w(\mathit{isList})\\
&\quad = \{(\rho\colon w \to w', (\underline{\mathsf{Ref}_{\mathsf{list}}}\rho)(\ell), \delta \in \hat{H}w')_\sim \mid \mathsf{pr}_{\rho(\ell)}(\delta) = \mathit{null}\} \cup\\
&\qquad\quad [\![\ell\colon \mathsf{Ref}_{\mathsf{list}}, \mathit{isList}\colon \mathsf{P}(\mathsf{Ref}_{\mathsf{list}}) \vdash \exists \ell', x.\, \ell \hookrightarrow (x,\ell') \star \mathit{isList}(\ell')]\!]_w(\mathit{isList})\\
&\quad = \{(\rho\colon w \to w', (\underline{\mathsf{Ref}_{\mathsf{list}}}\rho)(\ell), \delta \in \hat{H}w')_\sim \mid\\
&\qquad\quad \mathsf{pr}_{\rho(\ell)}(\delta) = \mathit{null} \;\vee\; \exists \ell',\; x.\; \mathsf{pr}_{\rho(\ell)}\,\delta = (x,\ell') \wedge (\rho, \ell', \delta \setminus \rho(\ell))_\sim \in \mathit{isList}\}
\end{aligned}$$

where $\delta \setminus \rho(\ell)$ denotes the $\delta$ with the cell $\rho(\ell)$ removed. In summary, $(\rho\colon w \to w', (\underline{\mathsf{Ref}_{\mathsf{list}}}\rho)(\ell), \delta \in \hat{H}w')_\sim$ is in $[\![\ell\colon \mathsf{Ref}_{\mathsf{list}}, \mathit{isList}\colon \mathsf{P}(\mathsf{Ref}_{\mathsf{list}}) \vdash \mathit{isList}(\ell)]\!]_w(\mathit{isList})$ if and only if either $\mathsf{pr}_{\rho(\ell)}\,\delta = \mathit{null}$ or there exists an $l' \in w'$ such that $\mathsf{pr}_{\rho(\ell)}\,\delta = (x,\ell')$ and $(\rho, \ell', \delta \setminus \rho(\ell))_\sim \in \mathit{isList}$.

## 8 Conclusions and Further Work

Compositionality is an uncontroversial desirable property in semantics and reasoning, which admits strikingly different, but equally valid interpretations, as becomes particularly instructive when modelling dynamic memory allocation. From the programming perspective it is desirable to provide compositional means for keeping track of integrity of the underlying data, in particular, for preventing *dangling pointers*. Reasoning however inherently requires introduction of partially defined data, such as *heaplets*, which due to the compositionality principle must be regarded as first class semantic units.

Here we have made a step towards reconciling recent extensional monad-based denotational semantic for full-ground store [9] with higher order categorical reasoning frameworks [2] by constructing a suitable intuitionistic BI-hyperdoctrine. Much remains to be done. A highly desirable ingredient, which is currently missing in our logic in Fig. 4 is a construct relating programs and logical assertions, such as the following dynamic logic style modality

$$\frac{\mathit{\Gamma} \vdash_{\mathsf{c}} p\colon A \qquad \mathit{\Gamma} \vdash \phi\colon \mathsf{P}A}{\mathit{\Gamma} \vdash [p]\phi\colon \mathrm{prop}}$$

which would allow us e.g. in a standard way to encode *Hoare triples* $\{\phi\}p\{\psi\}$ as implications $\phi \Rightarrow [p]\psi$. This is difficult due to the outlined discrepancy in the semantics for construction and reasoning. The categories of initializations for $p$ and $\phi$ and the corresponding hiding monads are technically incompatible. In future work we aim to deeply analyse this phenomenon and develop a semantics for such modalities in a principled fashion.

Orthogonally to these plans we are interested in further study of the full ground store monad and its variants. One interesting research direction is developing algebraic presentations of these monads in terms of operations and equations [17]. Certain generic methods [13] were proposed for the simple store case (Example 3), and it remains to be seen if these can be generalized to the full ground store case.
